# Anticoagulant Rodenticides on our Public and Community Lands: Spatial Distribution of Exposure and Poisoning of a Rare Forest Carnivore

**DOI:** 10.1371/journal.pone.0040163

**Published:** 2012-07-13

**Authors:** Mourad W. Gabriel, Leslie W. Woods, Robert Poppenga, Rick A. Sweitzer, Craig Thompson, Sean M. Matthews, J. Mark Higley, Stefan M. Keller, Kathryn Purcell, Reginald H. Barrett, Greta M. Wengert, Benjamin N. Sacks, Deana L. Clifford

**Affiliations:** 1 Integral Ecology Research Center, Blue Lake, California, United States of America; 2 Veterinary Genetics Laboratory, University of California Davis, Davis, California, United States of America; 3 California Animal Health and Food Safety Laboratory System, University of California Davis, Davis, California, United States of America; 4 Sierra Nevada Adaptive Management Project, University of California, Berkeley, California, United States of America; 5 Pacific Southwest Research Station-Sierra Nevada Research Center, United States Forest Service, Fresno California, United States of America; 6 Wildlife Conservation Society, Hoopa, California, United States of America; 7 Wildlife Department, Hoopa Tribal Forestry, Hoopa, California, United States of America; 8 Department of Pathology, Microbiology and Immunology, University of California Davis, Davis, California, United States of America; 9 Wildlife Investigations Laboratory, California Department of Fish and Game, Rancho Cordova, California, United States of America; University of California, Berkeley, United States of America

## Abstract

Anticoagulant rodenticide (AR) poisoning has emerged as a significant concern for conservation and management of non-target wildlife. The purpose for these toxicants is to suppress pest populations in agricultural or urban settings. The potential of direct and indirect exposures and illicit use of ARs on public and community forest lands have recently raised concern for fishers (*Martes pennanti*), a candidate for listing under the federal Endangered Species Act in the Pacific states. In an investigation of threats to fisher population persistence in the two isolated California populations, we investigate the magnitude of this previously undocumented threat to fishers, we tested 58 carcasses for the presence and quantification of ARs, conducted spatial analysis of exposed fishers in an effort to identify potential point sources of AR, and identified fishers that died directly due to AR poisoning. We found 46 of 58 (79%) fishers exposed to an AR with 96% of those individuals having been exposed to one or more second-generation AR compounds. No spatial clustering of AR exposure was detected and the spatial distribution of exposure suggests that AR contamination is widespread within the fisher’s range in California, which encompasses mostly public forest and park lands Additionally, we diagnosed four fisher deaths, including a lactating female, that were directly attributed to AR toxicosis and documented the first neonatal or milk transfer of an AR to an altricial fisher kit. These ARs, which some are acutely toxic, pose both a direct mortality or fitness risk to fishers, and a significant indirect risk to these isolated populations. Future research should be directed towards investigating risks to prey populations fishers are dependent on, exposure in other rare forest carnivores, and potential AR point sources such as illegal marijuana cultivation in the range of fishers on California public lands.

## Introduction

Anticoagulant rodenticide (AR) exposure and poisoning has emerged as a conservation concern for non-target wildlife [1], [2], [3]. These toxicants are used to eradicate or suppress rodent pest populations in agricultural or urban settings to minimize economic losses [1], [4]. Generally, the mechanism of AR function is to bind and inhibit enzyme complexes responsible for the recycling of vitamin K_1_, thus creating a series of deleterious clotting and coagulation impairments [4], [5]. The ARs are grouped into two classes: first-generation compounds, which require several doses to cause intoxication, and second-generation ARs, which are more acutely toxic often requiring only a single dose to cause intoxication and persist in tissues and in the environment [1], [4], [6], [7]. Rodents have started to develop resistance to both first-generation and second-generation ARs, prompting increasingly greater reliance on more acutely toxic compounds and increased distribution by AR users [1], [7], [8].

Primary exposure by ingestion of bait or secondary exposure through consumption of exposed prey has been documented in numerous species of endangered and common non-target wildlife [1], [3], [9], [10], [11], [12], [13]. Wildlife are thought to be at greatest risk of exposure to ARs in agricultural, urban or peri-urban settings, where large quantities of these compounds are often used [12], [14], [15]. However, little is known about the risks to wildlife in settings with little or no anthropogenic influences.

**Figure 1:**
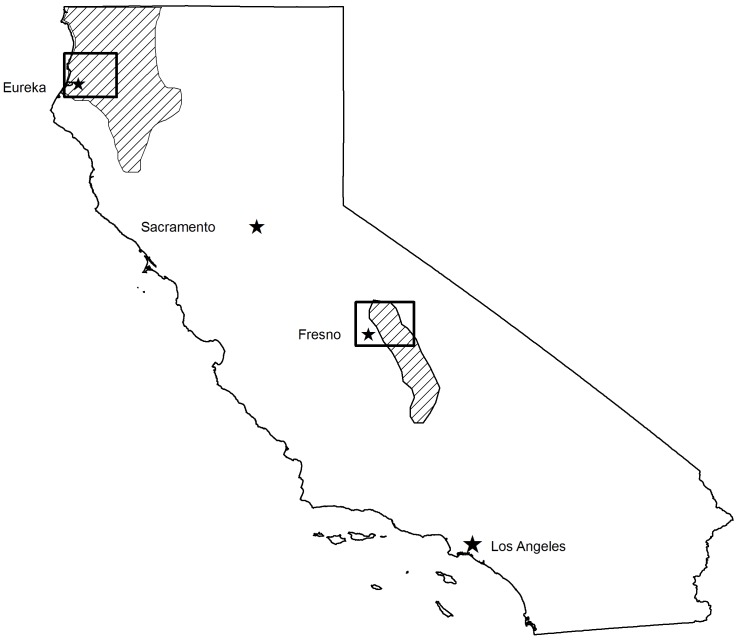
Fisher (*Martes pennanti*) current range in California and project areas. Current range (shaded areas) of the two isolated California populations of fishers (*Martes pennanti*). Areas of fisher projects that generated data for exposure and mortality to anticoagulant rodenticides are outlined within the two isolated populations.

Fishers (*Martes pennanti*), a large mustelid and the largest member in the genus Martes, were once widely distributed throughout west coast of North America, but have experienced significant population declines, including extirpation from some regions and contractions of historic ranges [16], [17], [18]. Populations of fishers inhabiting California, Oregon and Washington have been designated as a Distinct Population Segment (DPS) and declared a candidate species for listing under the federal Endangered Species Act [17], [19]. The west coast DPS encompasses areas where fishers were extirpated from Washington and central and northern Oregon, a reintroduced population in the Cascade mountains of southern Oregon, and two extant and isolated populations, one spanning southern Oregon and northern California and another in the southern Sierra Nevada mountains of California [17], [19]. The population status of fishers in the southern Oregon/northern California is unknown; however population estimates for the isolated fisher population in the southern Sierra Nevada range from 150–300 fishers, with 120–250 in the adult age class [17], [20], [21]. Because fishers in the DPS occur in and are dependent on mid to late-seral stage coniferous and hardwood forests and are not associated with agricultural or urban settings, toxicants have not been previously considered a likely threat to fisher populations [17], [22], [23].

**Figure 2:**
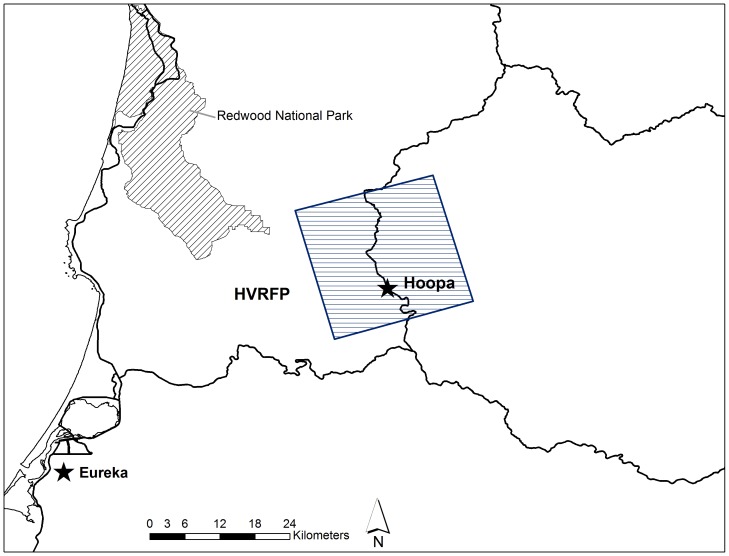
Enlarged map of fisher (*Martes pennanti*) project area for the northern California population at the Hoopa Valley Reservation Fisher project (HVRFP).

We assessed the magnitude of AR exposure and poisoning among fisher carcasses submitted for necropsy from 2006 to 2011 as part of a collaborative effort studying threats to population persistence of fishers in California. Additionally, spatial analysis of telemetry data from sampled fishers was conducted in an effort to identify potential sources of AR in the environment. We hypothesized that due to fishers being a forest-dependent carnivore, exposure to ARs will be rare.

**Figure 3:**
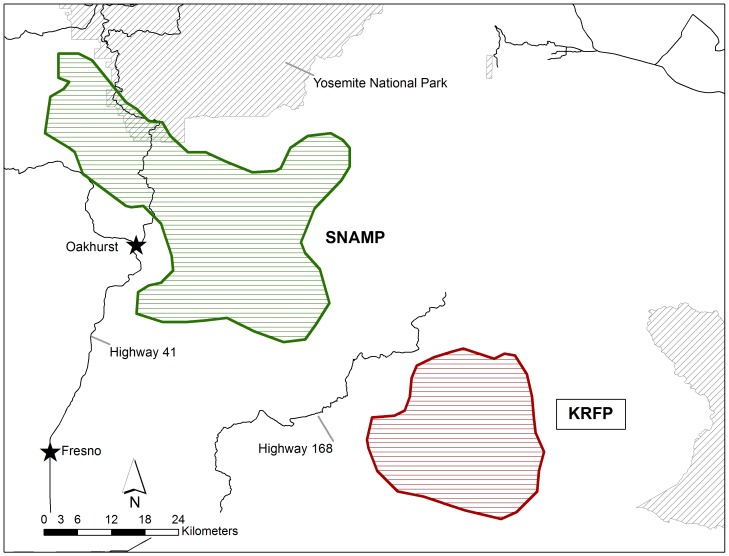
Enlarged map of fisher (*Martes pennanti*) project areas for the southern Sierra Nevada population: the Sierra Nevada Adaptive Management Project (SNAMP) and Kings River Fisher Project (KRFP).

**Table 1:** Exposure and mortality due to anticoagulant rodenticides (AR) fishers (*Martes pennanti*) within the two isolated populations, northern California and southern Sierra Nevada.

Fisher population	Number of fisherstested (F:M)	Number of AR exposed fishers (F:M)	Number of AR mortalities	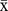 , SD and range in all, female (F) andmale (M) exposed fishers	Chi-square	Probability Level	DF
Northern California	18 (11∶7)	13 (72%) (8∶5)	2	1.38 (SD = 0.84; range1–3) (F) 1.13 (SD = 0.35;range 1–3) (M) 1.8 (SD = 0.84; range 1–3)	0.004	0.952	1
Sierra Nevada	40 (18∶22)	33 (83%) (16∶17)	2	1.70 (SD = 0.88; range1–4) (F) 1.47 (SD = 0.87;range 1–4) (M) 2.00 (SD = 0.85; range 1–4)	0.925	0.336	1
All California	58 (31∶27)	46 (79%) (26∶20)	4	1.61 (SD = 0.83; range 1–4) (F) 1.33 (SD = 0.73;range 1–4) (M) 2.00 (SD = 0.82; range 1–4)	0.844	0.358	1
Heterogeneity chi-square					0.085	0.77	1

Mean number (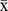 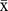) of AR compounds detected per individual, range of numbers of AR per individual and standard deviation (SD) are given for all, female (F) and male (M) fishers for each population. Chi-square and heterogeneity Chi-square test analyzing exposure between the sexes both within and between the populations.

**Figure 4:**
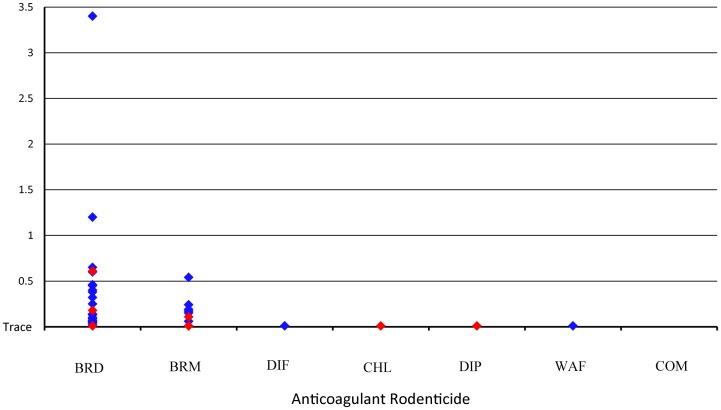
Quantification levels of anticoagulant rodenticides detected in California fishers. Anticoagulant rodenticides (AR) brodifacoum (BRD), bromodiolone (BRM), difethialone (DIF), chlorophacinone (CHL), diphacinone (DIP), warfarin (WAF) and coumachlor (COM) parts per million (PPM) levels detected in positive fishers (*Martes pennanti*) in California. Blue diamonds represent AR quantification levels (ppm). Red diamonds represent levels in fishers that died due to AR ingestion.

## Methods

### Ethics Statement

All procedures involving animals were reviewed and approved by the University of California, Davis, Animal Care and Use Committee (Protocol No. 16551).

**Figure 5:**
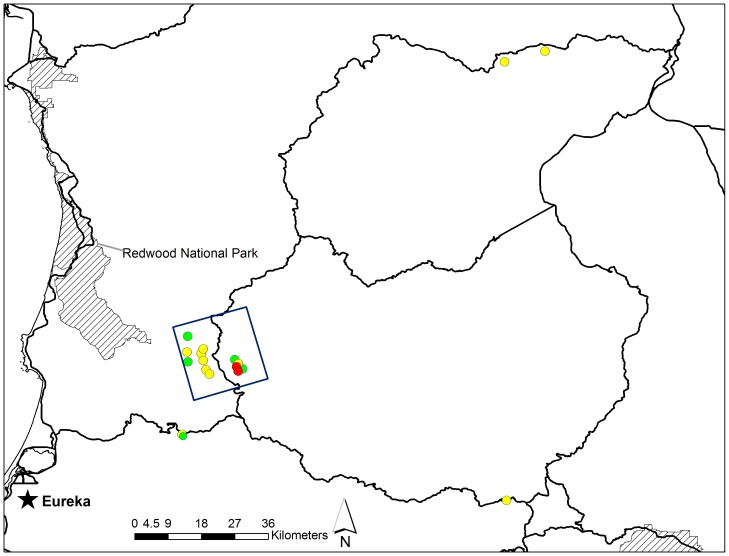
Exposure to and mortality from anticoagulant rodenticides (AR) in fishers (*Martes pennanti*) from the isolated northern California population. Green circles represent negative fishers, yellow circles represent exposed fishers, while red circles are fishers that died due to AR toxicosis.

**Figure 6:**
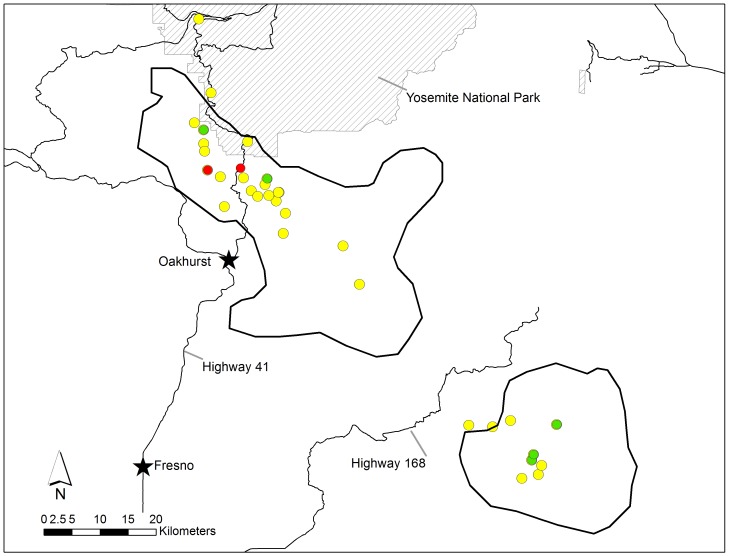
Exposure to and mortality from anticoagulant rodenticides (AR) in fishers (*Martes pennanti*) from the isolated southern Sierra Nevada population. Green circles represent negative fishers, yellow circles represent exposed fishers, while red circles are fishers that died due to AR toxicosis.

### Study Area

Fishers were captured in box traps modified with a plywood cubby box (model 207, Tomahawk Live Trap Company, Tomahawk, Wisconsin, USA), sampled, and fitted with a VHF radio-collar and monitored via telemetry. Fisher carcasses were submitted from the two isolated California populations by three fisher monitoring projects (Figure 1). Carcasses from the northern California population were submitted by the Hoopa Valley Reservation Fisher Project (HVRFP), conducted in northwestern California within tribal, private and public lands, and non-monitored fishers on public and private lands throughout the northern Sierra Nevada/southern Cascade Mountain borderlands of north central California (Figure 2). Carcasses from the southern Sierra Nevada California population were submitted by the Sierra Nevada Adaptive Management Project (SNAMP) and the USDA Forest Service Kings River Fisher Project (KRFP); both projects were conducted on the Sierra National Forest in the northern and central portions of this population’s extent (Figure 3).

### Sample Collection

Deceased fishers were collected by project personnel whenever a fisher was determined to be inactive for >24 hours, a mortality signal from the VHF collar was detected or when unmarked fisher carcasses were opportunistically observed at the project sites or adjacent areas. Fisher carcasses were stored in a −20 °C freezer until a complete necropsy to determine causes of mortality was performed by a board-certified pathologist specializing in wildlife at the California Animal Health and Food Safety Laboratory System (CAHFS) or the University of California Davis Veterinary Medical Teaching Hospital in Davis, CA, USA. Liver samples were collected during necropsy and submitted for screening and quantification of seven ARs at CAHFS by liquid chromatography-tandem mass spectrometry for screening presence of ARs and high-performance liquid chromatography to quantitate positive samples. The AR compounds tested for included first-generation ARs, warfarin (WAF), diphacinone (DIP), chlorophacinone (CHL), and coumachlor (COM); and second-generation ARs, brodifacoum (BRD), bromodiolone (BRM), and difethialone (DIF). The reporting limits were 0.01 ppm for BRD, 0.05 for WAF, BRM, and COM, and 0.25 ppm for DIP, CHL, and DIF. Detectable compound concentrations that were below quantitate limits were labeled as “trace” concentrations. All results were reported on a tissue wet weight basis and reviewed by a board-certified toxicologist [12], [24].

**Figure 7:**
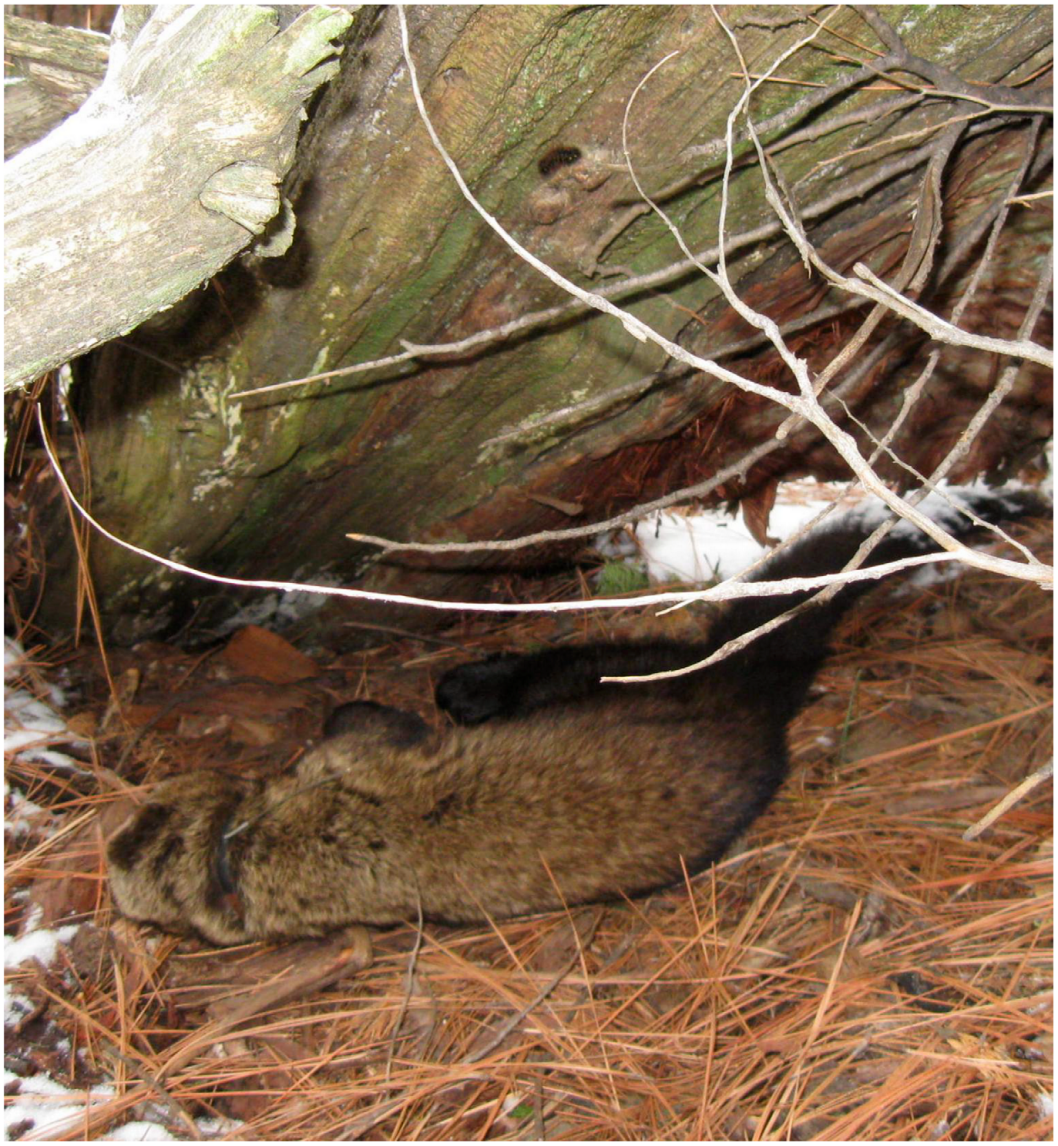
Condition of the undisturbed mortality site in which a fisher (*Martes pennanti*) mortality due to anticoagulant rodenticide from the southern Sierra Nevada population was found.

**Figure 8:**
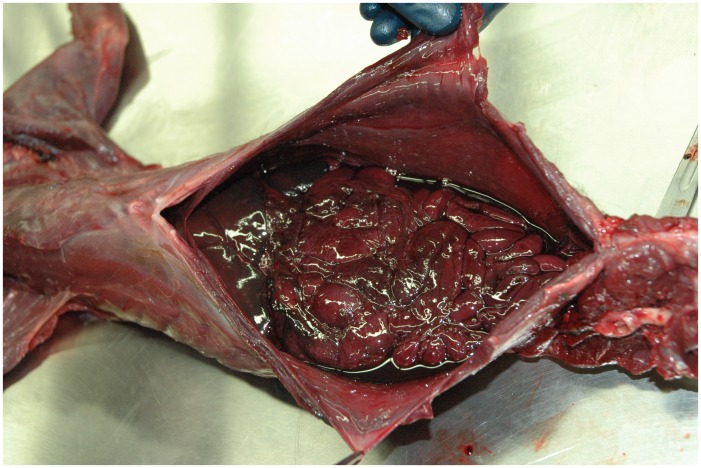
Thoracic cavity hemorrhaging containing 150 ml of frank blood due to coagulopathy after lethal exposure to anticoagulant rodenticides in a fisher (*Martes pennanti*) from the southern Sierra Nevada population.

Age classification was determined by tooth wear, sagittal crest or testicular/teat development, field and laboratory observation, and monitoring of individual animals [17], [18], [25]. Fishers were classified as kits when fully or semi-altricial and dependent on milk for nourishment (roughly ≤10 weeks), juveniles if weaned and <12 months of age, sub-adults when between 13–24 months of age, and adults ≥24 months of age [17], [18], [25].

### Statistical Analysis

Prevalence of AR exposure among fishers was calculated for the total sample, each sex and each age class. We compared the AR exposure prevalence between sexes within and between the two California populations using two-tailed heterogeneity chi-square tests of association [26]. The effects of sex and population on the number of anticoagulant rodenticides found per individual were analyzed with a two-way ANOVA [27]. All tests were conducted using the program NCSS (Number Cruncher Statistical Software, Kaysville, UT, USA) with an alpha level *p* = 0.05.

### Spatial Analysis

For monitored fishers, telemetry locations were used to generate 95% minimum convex polygon (MCP) home-range centroids to represent a centralized point within the core area of movement within each individual fisher home-range within each project area [28]. For each fisher, three centroids representing three sampling timeframes were calculated using ArcView 9.1 home range extensions (ESRI Inc., Redlands CA., USA) [29]. The first centroid incorporated all fisher locations from initial capture until death, irrespective of the monitoring time; the second centroid incorporated fisher locations collected six months prior to death; and the third centroid incorporated only the fisher locations collected three months prior to death. These two latter centroids containing locations collected over a shorter time period prior to death were calculated because some ARs have relatively short half-lives and any spatial clustering in these MCP centroids might suggest the locale of recent sources of AR exposure. Only fishers with ≥3 months of monitoring were used for spatial analysis, individuals that had less than or were opportunistically collected were excluded.

**Figure 9:**
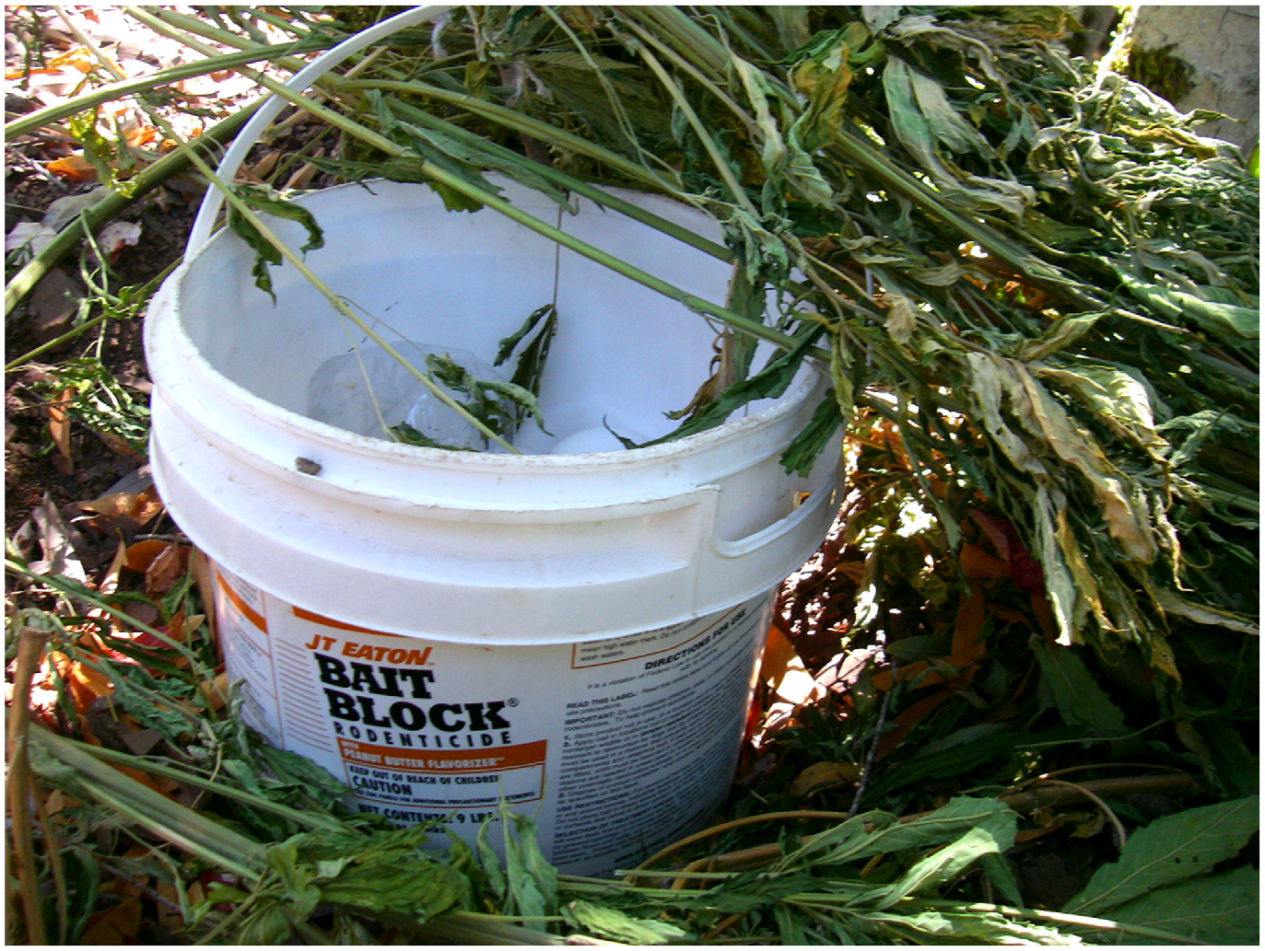
One of several nine-pound buckets of anticoagulant rodenticide removed from an illegal northern California marijuana operation within the northwestern California fisher (*Martes pennanti*) project boundary.

**Figure 10:**
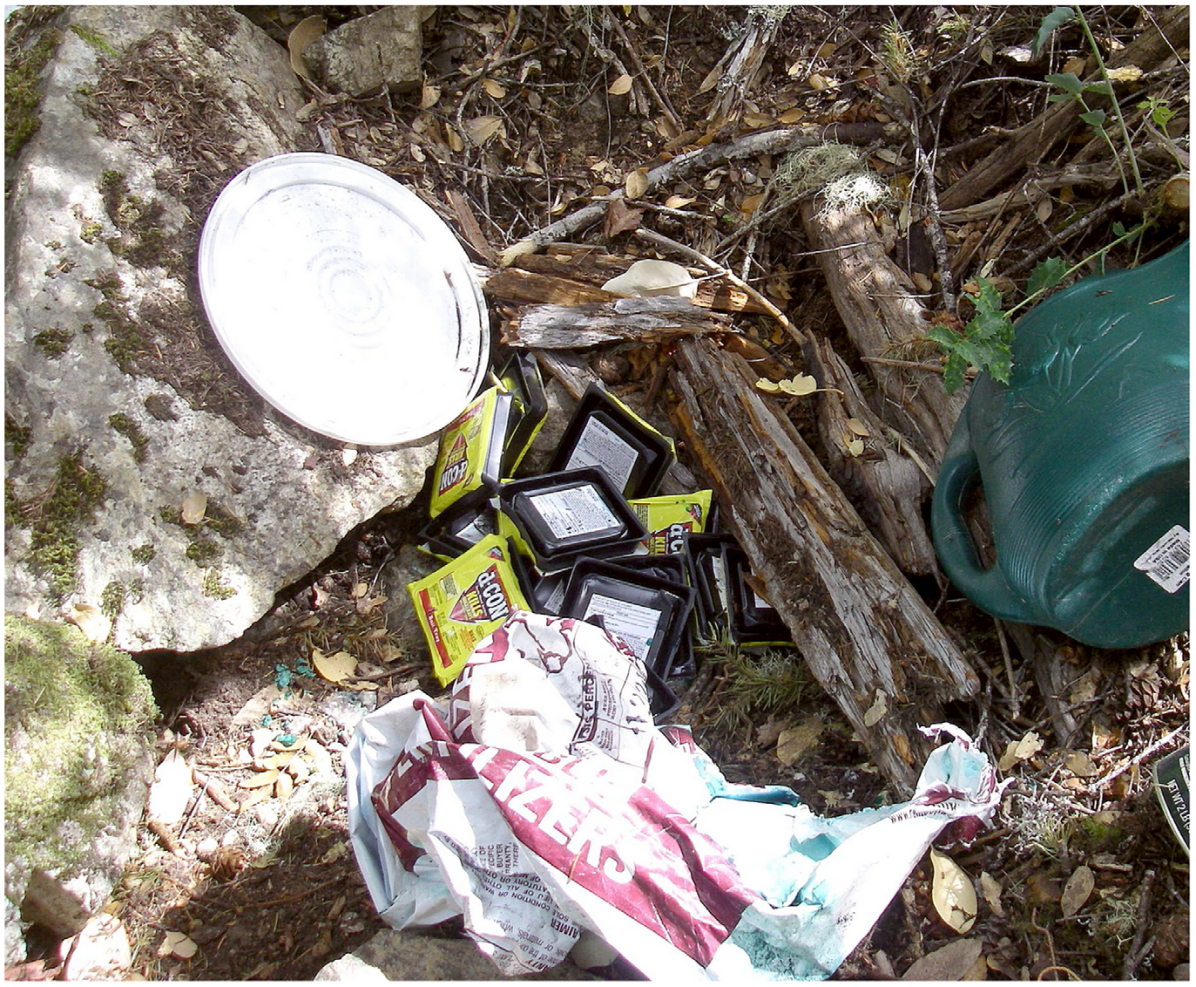
Multiple packets of anticoagulant rodenticides found surrounding an illegal marijuana grow site within the southern Sierra Nevada fisher (*Martes pennanti*) project.

**Figure 11:**
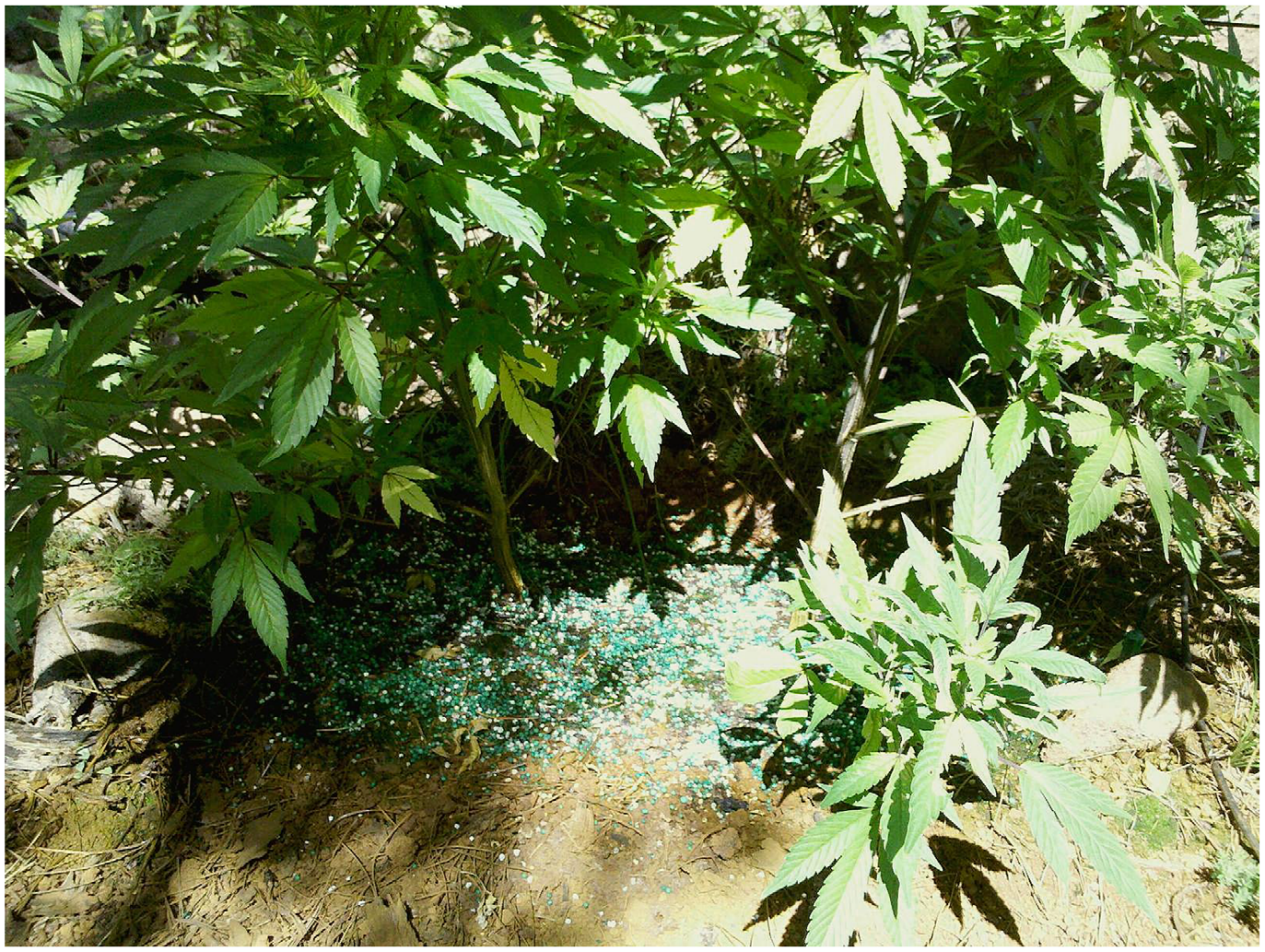
Anticoagulant rodenticide bait pellets (bright green) with plant fertilizer freely dispersed around 2,000 plants from northern California marijuana grow site within the northwestern California fisher (*Martes pennanti*) project boundary.

Centroids were analyzed by spatial scan statistics to determine whether exposure to ARs, exposure to different generation classes (1^st^ and 2^nd^) of ARs, or exposure to individual compounds of ARs were distributed uniformly or spatially clustered in each of the two California populations [30]. SaTScan version 9.1.1 (M. Kulldorff, Harvard Medical School, Boston, MA, USA) was used to evaluate two separate models. First, a Bernoulli model utilizing count data was used to determine if spatial clustering occurred in exposed and non-exposed fishers, or in first or second-generation class AR exposure. The second model, a multinominal model using categorical data, was used to assign each fisher to a group based on the number of AR compounds detected and to examine possible clustering of individuals with high numbers of AR compounds [31]. SatScan uses these models to scan the geographic area encompassing the MCP centroids to detect spatial clusters encompassing not more than 50% of the centroids [32]. The elliptical scanning window option was chosen for both models because it utilizes both circular and elliptical shapes to allow for a better fit to linear geographic features (i.e. drainages or ridgelines) that occur within the fisher’s habitat [32], [33]. All statistical values from the models were generated by Monte Carlo simulations of 999 iterations and clusters evaluated for significance with alpha = 0.05.

## Results

### Population-level Exposure to AR

Forty-six of the 58 fisher carcasses tested (79%) were exposed to one or more compound of AR (Table 1). Frequency of exposure (p>0.05) and the number of ARs per fisher (p>0.05) were similar between populations and sexes (Table S1). The number of AR compounds detected per individual ranged from 1–4 (Table 1). Exposure to at least one AR among age classes ranged with one of 4 pre-weaned kits (25%), 4 of 4 (100%) juveniles, 12 of 17 (70%) sub-adults, and 29 of 33 (88%) adults. Both first and second generation ARs were detected, with BRD being most common and detected in 44 of the 46 (96%) exposed fishers, followed by BRM (16 of 46; 35%), DIP (8 of 46; 17%), CHL (four of 46; 9%), DIF (one of 46; 2%), and WAF (one of 46; 2%). Quantifiable levels of BRD (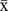 = 0.22 ppm; range trace −3.4 ppm) and BRM (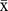 = 0.12 ppm; range trace −0.54 ppm) were detected while only trace levels of other ARs were detected (Figure 4). No samples had detectable levels of COM and no indicator dye or AR bait was detected in either stomach or the GI contents of any fisher.

### Northern California Fishers

Thirteen of 18 (72%) fishers from the northern California population were exposed to an AR compound (Table 1). Brodifacoum was detected in 12 (92%), BRM in two (15%), DIP in two (15%), CHL in one (8%), and WAF in one (8%) of the 13 exposed individuals.

### Sierra Nevada Fishers

Thirty-three of 40 (83%) fishers from the southern Sierra Nevada were exposed to an AR compound (Table 1). Brodifacoum was detected in 32 (97%), BRM in 14 (42%), DIP in six (18%), CHL in three (9%), and DIF in one (3%) of the 33 exposed individuals.

### Spatial Distribution of AR Exposure

Complete centroids were generated for 42 monitored fishers, 12 fishers from the northwestern California population (all 12 from HVRFP) and 30 from the southern Sierra Nevada population (19 from SNAMP, 11 from KRFP). Of these fishers, 3-month MCP centroids were generated for 39 fishers, and 6-month centroids for 27 (Table S2). Spatial analysis for 6-month centroids from the KRFP could not be conducted because all fishers in the data set were AR exposed. Sixteen fishers were excluded from the analysis due to lack of monitoring data. No spatial clustering of AR exposure was detected for any of the temporal periods, specific AR compounds, generation class of AR, or distribution of numbers of ARs per fisher in any of the study areas (Table S2; Figure 5, Figure 6).

### AR-Mortalities

Cause-specific mortality factors for all 58 fishers sampled ranged widely and included predation, infectious and non-infectious disease processes and vehicular strikes (M.W. Gabriel unpublished data). The cause of death for four of these fishers was attributed to lethal toxicosis, indicated by AR exposure with simultaneous coagulopathy and bleeding into tissues or cavities and ruling out any concurrent processes that might cause hemorrhaging [34]. Two of the four fishers killed by ARs were from the southern Sierra Nevada population, and two were from northern California (Table 1) and the case details are described below.

### Southern Sierra Nevada

An adult male fisher was recovered on 15 April 2009, in the southern Sierra Nevada at the SNAMP project area. The fisher showed no signs of predation or scavenging (Figure 7). Gross necropsy determined that the fisher was in good nutritional (3.45 kg) and fair postmortem condition. Frank blood was observed in both the thoracic and abdominal cavities (150 ml and 100 ml respectively), and in the pericardial sac (7 ml) (Figure 8). The stomach and lower gastrointestinal tract contained some blood but no prey or formed feces, and no mucosal changes were noted. There were no other findings on gross examination. Histopathologically, no significant changes were observed in any tissues. Brodifacoum and BRM were detected and quantified in the liver sample at 0.38 ppm and 0.11 ppm, respectively, and CHL at trace levels (Figure 4).

The second fisher mortality was a lactating adult female recovered on 2 May 2010 in the center of a paved rural highway in the SNAMP project area approximately 3.7 km from Yosemite National Park. Vehicular strike was initially suspected as the cause of mortality due to the location of the carcass but lacerations, abrasions and visual evidence of trauma were not seen on gross examination of the intact carcass. The post-mortem state of the carcass was good and the nutritional state was poor (2.54 kg). Shallow subcutaneous hemorrhage was noted over the hindquarters and spinal column with no associated fractures, punctures or abrasions. There was approximately 20 ml of frank blood within the thoracic cavity. There was no evidence of pneumothorax, vessel ruptures, or visceral tearing. No blood or visceral damage was seen in the abdominal cavity. Stomach contents contained various rodent parts with formed feces in the descending colon. Histopathologically, no significant changes were observed in any tissues. Brodifacoum and BRM were detected and quantified at 0.60 ppm and 0.17 ppm, while one first generation AR, DIP was detected at a trace level within the liver tissue (Figure 4). No evidence was present to suggest that this fisher died due to vehicular trauma, despite its location on the highway.

### Northern California

A sub-adult male fisher was recovered on 4 May 2010 at the base of several riparian shrubs near a watercourse in northwestern California at the HVRFP. Severe ectoparasitism on the carcass was noted in the field with ticks in both replete and non-replete stages. Predation was not suspected due to absence of external wounds. The gross necropsy determined that this fisher (2.65 kg) was in poor nutritional condition with no subcutaneous or visceral fat. Frank blood was present in the right external ear canal, nasal and oral cavities, within the lumen of the trachea and within the periorbital tissue with no associated skull fractures or punctures. The stomach was devoid of prey. The colon only contained semi-formed feces. Ectoparisitism was severe with approximately 48 female and 10 male American dog ticks (*Dermacentor variabilis*) and 8 female and 2 male western black-legged ticks (*Ixodes pacificus*) removed from various regions of the fisher. The liver sample from this fisher had quantifiable levels of BRD at 0.04 ppm as well as a trace level of CHL (Figure 4).

The second northern California fisher AR death, was an adult male recovered on 26 May 2010 at the HVRFP. Field observations included no evidence of predation or scavenging. The nutritional state as well as the postmortem condition were poor. Gross necropsy determined that the fisher (2.89 kg) had no body fat present in any of the tissues. Frank blood was present in both thoracic and abdominal cavities. The stomach contained red and black fluid but no prey. Ectoparasitism was severe with 204 female and 27 male adult American dog ticks in both replete and non-replete stages on areas of the muzzle, chest, tops of fore-and hind-limbs as well as inguinal sections. Severe nematodiasis was seen in skeletal muscle throughout the body (trichinosis). Pulmonary nematodiasis (lungworm) was also noted in the marginal portions of the lungs. Histopathologically, no notable disease processes were seen but severe parasitism was noted. The liver sample for this fisher had quantifiable levels of BRD at 0.61 ppm and trace levels of BRM (Figure 4).

### Neonatal Transfer of AR

Necropsies and AR testing was performed on four kits who were all still dependent on mother’s milk when they died following maternal abandonment from their mothers death. One kit, a female fisher (0.32 kg) from KRFP tested positive for AR exposure. This kit was approximately six weeks of age and was recovered within a monitored maternal den tree shortly after maternal abandonment. Cause of death was determined to be acute starvation and dehydration. The liver tissue contained trace level of BRD but there was no associated hemorrhaging in any tissues, body cavities or lumina, suggesting that this finding was not clinically significant.

## Discussion

Our findings demonstrate that anticoagulant rodenticides, which were not previously investigated in fishers or other remote forest carnivores, are a cause of mortality and may represent a conservation threat to these isolated California populations. This is the first documentation of exposure to ARs and of direct mortality from ARs in fishers anywhere in their geographic range. Earlier studies suggest ARs posed little or no additive mortality effects on non-target populations [7], [35], [36]. The shortfall of many of these studies was the utilization of common cosmopolitan species so they did not take in consideration that AR mortality may be additive in otherwise compromised populations. The spatially ubiquitous exposure observed within all post-weaning age classes and across the project areas in their contemporary range in California is of significant concern especially considering the recent work of Spencer et al. (2010), who demonstrated that even a small increase in human-caused mortality of 10–20% in the isolated Southern Sierra Nevada fisher population would be enough to prevent population expansion if other restrictive habitat elements were removed.

The high rate of exposure to second generation AR compounds (96% of exposed fishers) in these populations is surprising and cause for concern. This generation of ARs are not only more acutely toxic, but have long retention (>150 days half-life) through biphasic elimination in mammal tissues [1], [37]. Second-generation ARs are more toxic because death can occur from a single primary ingestion by a rodent [1], [5], [37], [38]. However, rodents can receive a lethal dose of second-generation ARs in one feeding bout and it can take up to 7 days before clinical signs manifest [1], [39]. Therefore, prey that have consumed a “super-lethal” dose of AR can pose a substantial risk to predators for several days prior to death [39]. In one study, a group of Norway rats (*Rattus norvegicus*) was given a choice between BRD bait and untreated food and another group had access only to the BRD bait [1]. Both groups consumed 10 and 20 median lethal doses (LD_50_) on the first day and 40 to 80 LD_50_ doses by day 6.5, respectively [1]. If sources for these toxicants are maintained for even short periods, exposed rodents, the main prey source for fishers in these populations [17] can pose significant threats to their predators.

Many manufactures use “flavorizers” since the AR compound may be bitter and unpalatable to rodent pests [1], [39]. Emulsions used to increase palatability include sucrose, bacon, cheese, peanut butter, and apple flavors (Sure-Gro Inc., Brantford, Ontario, Canada and J.T. Eaton, Twinsburg, Ohio, USA), and thus could be palatable to generalist carnivores like fishers. Although we did not visually detect AR bait in the stomach or GI tracts of any fishers that died, primary poisoning cannot be completely ruled out.

### Sub-lethal AR Exposure

In addition to the risk from lethal toxicosis, sub-lethal AR exposure may compromise fishers through a reduction in the function of normal clotting [5], [37], [40], [41]. The occurrence of AR -exposed wildlife dying from minor wounds that otherwise might have easily resolved themselves if ARs were not present suggests contributory lethal effects [1]. Several cases describe raptors receiving minor defensive lacerations or trauma from prey that lead to the raptor’s death by exsanguination or hemorrhaging [1], [42]. Fishers actively pursue a wide array of terrestrial and arboreal prey [17], [18]. Hence, it is conceivable that a fisher could receive similar wounds or trauma from prey, or during the pursuit of prey. Consequently, if clotting mechanisms were compromised due to ARs, benign injuries could lead to serious complications [1], [42], [43], [44]. The leading causes of mortality within the USFWS DPS is intraguild predation (G.M.Wengert, unpublished data). It is possible that some of these cases, AR exposure could have compromised clotting mechanisms at the predation attempt and this deserves further study.

High levels of tick infestations were noted in two of the AR mortalities when compared to other sympatric species within the same project area [45]. In addition, locations of of these replete ticks were in infrequent regions in other captures, most likely due to a lack of regular grooming. Whether ARs played a role by allowing more ticks to obtain a blood meal due to immobilization due to compromised clotting factors is unknown.

Furthermore, sublethal AR exposure may decrease an animal’s resilience to environmental stressors. In a study on rabbits and rats subjected to stressors such as severe decreases in ambient temperature (i.e. frostbite), approximately 10% of test animals died; however when animals were exposed to low non-lethal doses of anticoagulants and subjected to the same stressors, mortality rates increased to 40–70% [46]. It is unknown if stressors or injuries from environmental, physiological or even pathogenic factors could predispose fishers to elevated mortality rates when coupled with AR exposure.

### Neonatal Transfer of AR

The documentation of neonatal or lactational transfer of AR to a dependent fisher kit was unexpected, and the effects of AR exposure to a kit during fetal development or shortly after birth are unstudied. AR exposure in pregnant or whelping domestic canids varied, causing no clinical signs in some cases [47] but death due to coagulopathy immediately after delivery in other cases [48]. The female fisher who gave birth to this kit did not exhibit clinical signs at pre- or postpartum captures and monitoring of her maternal den site verified that one kit survived from that litter (Rebecca Green, United States Forest Service, personal communication). Nevertheless, clinical signs including hemorrhaging, inappetence and lethargy have been seen in domestic canid puppies of AR-exposed mothers [47], [48]. Mild to severe manifestations such as low birth weight, stillbirth or eventually neonatal death has been documented in several cases [47], [48], [49]. In one human study where pregnant women received low doses of warfarin due to severe risk of thromboembolic events, 33% of them had stillbirths, 28% had abortions, and 11% of the neonates died shortly after birth [50]. The range for congenital anomalies and miscarriages in pregnant females for prescribed doses of warfarin varied from 15 to 56% and long-term neurological symptoms have been reported in children that were exposed in-utero [51]. The fetotoxic effects of AR in pregnant fishers and their fetuses are unknown. In addition, because fishers exhibit delayed implantation of the blastocyst, whether ARs may cause pregnant females to abort or reabsorb the fetus merits further research [52], [53], [54]. The transfer of first generation ARs from mother to offspring in milk is not well-understood and there are no data on lactational transfer of second-generation ARs [49].

### Quantification Levels

The quantity (ppm) of AR we observed in fisher liver tissues varied and overlapped extensively in both sublethal and lethal cases with no clear indication of a numeric threshold that might indicate an amount leading to morbidity or mortality. This lack of predictive ability has been shown in numerous wildlife cases [1], [12], [55]. For example, Brodifacoum, the most prominent AR compound detected in fishers in this study ranged considerably in lethal cases among individual mustelid species, with 0.32–1.72 ppm in stoats (*Mustela ermine*) [55], [56], [57], 0.7 ppm in least weasels (*Mustela nivalis*) [56], 1.47–1.97 in ferrets (*Mustela furo*) [57] and 9.2 ppm in American mink (*Mustela vision*) [3], [36]. In addition, there are stark differences for acute LD_50_ doses among genera, where minute amounts of brodifacoum bait caused death in domestic canids but domestic felids required doses 5 to 40 times higher [38]. The same variability seen in both mustelids and other carnivores suggests that predicting clinical thresholds for fishers would be pre-mature [1], [58]. Furthermore, AR exposed fishers had an average of 1.6 AR types within their systems, and possible interaction effects from a combination of 2 or more AR compounds within a fisher and other species are entirely unknown [1], [37].

### Potential Sources of AR

Spatial analyses did not reveal any obvious point sources of AR exposure. Instead, these analyses suggested that exposure is widespread across the landscape. Previous studies expected that exposure to AR compounds would be clustered near areas of human activity or inhabitations and that exposure would not be common outside of these areas [1], [12], [14], [24]. Incongruously, data from this study refuted this hypothesis thus making the finding even more significant. Furthermore, these exposures occurred within a species that is not closely affiliated with urban, peri-urban or agricultural settings in which second-generation ARs typically are [1], [12], [14], [24]. Federal and state regulations for anticoagulant rodenticide usage are specific for both generations. Before the June 2011 Environmental Protection Agency (EPA) regulations [39], second generation class ARs could be purchased at local retailers, with recommendations for placement in weather- and tamper-resistant bait containers no more than 50 feet from any building [39]. However, since June 2011, second generation ARs have not been available to consumers at retail, but only at agricultural stores (farm, tractor or feed stores) with additional form and weight restrictions [39]. These newly passed regulations are aimed at further restriction of irresponsible and illegal use of ARs [39]. However, we would have expected that with either pre- or post-June 2011 regulations, second generation AR exposed fishers would have overlapped with urban, peri-urban, or agricultural environments. This pattern is acknowledged in several studies, such as Riley et al. (2007) where bobcat (*Lynx rufus*) and mountain lion (*Felis concolor*) total quantification levels of AR exposure were associated with human-developed areas. Numerous studies have documented that secondary poisoning cases are closely associated with recent agricultural or urban pest eradication efforts [1], [13], [14], [24].

The majority of habitat that fishers in California and fishers throughout the DPS currently and historically occupied is not within or near agricultural or urban settings [17]. Several fishers that were exposed had been monitored their entire lives and inhabited public or community lands where human structures are rare or non-existent (M. Higley, R. Sweitzer, C. Thompson unpublished data). Therefore, exposure from first or second-generation AR use at or within 50 feet of residential or agricultural structures and settings were considered unlikely due to fisher habitat requirements and general lack of association with humans. This suggests that wide-spread non-regulated use of second generation second generation ARs is occurring within the range of fishers in California, especially on public lands.

A likely source of AR exposure to fishers is the emerging spread of illegal marijuana cultivation within California public and private lands [59], [60]. In 2008 in California alone, over 3.6 million outdoor marijuana plants were removed from federal and state public lands, including state and national parks, with thousands of pounds of both pesticides and insecticides found at grow sites [59], [60], [61]. In 2011, a three week eradication operation of marijuana cultivation removed over 630,000 plants and 23,316 kg of trash including 68 kg of pesticides within the Mendocino National Forest in the northern California fisher populations range [17], [62]. Anticoagulant rodenticides and pesticides are typically dispersed around young marijuana plants to deter herbivory, [60], [62], [63] but significant amounts of AR compounds are also placed along plastic irrigation lines used to draw water from in order to deter rodent chewing [60], [62], [63] (M.W. Gabriel, personal observation). A recent example in which over 2,000 marijuana plants were removed less than 12 km from one of the project areas revealed that plants on the peripheral edges as well as nearby irrigation had large amounts of second generation AR placed (Figure 9, Figure 10, Figure 11). Finally, just within a single eradication effort, multiple kilometers (>40 km) of irrigation line within National Parks and Forests in California were removed [60], [62]. Placement of ARs at the grow sites and along irrigation lines which jut out great distances from the grow site itself may explain why there are no defined clusters of AR exposure.

It is noteworthy that the AR fisher mortalities we documented occurred in different areas of their California range but within a relatively short seasonal period between mid-April to mid-May. We cannot specify the exact explanation or source contributing to all AR mortalities that occurred within this short temporal period. This period is when females are providing for offspring as well as males searching for mates; however, preliminary spatial data for fishers in California document that females have more confined home-ranges during this period, while males have slightly larger home-ranges (S. Matthews, R. Sweitzer, unpublished data).

Additionally, several books available to the general public identify the optimal time for planting marijuana outdoors is during mid to late spring, and seedlings are especially vulnerable to rodent pests [64], [65], [66]. Of additional concern is that April to May is the denning period for female fishers and a time when fisher kits are entirely dependent on their mothers [17], [18]. The documentation of a lactating female mortality attributed to AR toxicosis during this period suggests that most likely kits would be abandoned and die from female mortalities during this time.

In conclusion, this study has demonstrated that fishers in the western DPS, which are of conservation concern and a candidate for protection under the Endangered Species Act, are not only being exposed to ARs, but ARs are a direct cause of mortality and indirect mortality (i.e. kit abandonment) in both of California’s isolated populations. Consequently, these toxicants may not only pose a mortality risk to fishers but could also pose significant indirect risks by depleting rodent prey populations upon which fishers depend. The lack of spatial clustering of exposed individuals suggests that AR contamination is widespread within this species’ range and illegal or irresponsible use of ARs continues despite recent regulatory changes regarding their use. Because we do not know the long-term ecological ramifications of these toxicants left on site long after marijuana grows are dismantled, heightened efforts should be focused on the removal of these toxicants at these and adjacent areas at the time of dismantling. Further regulation restricting the use of ARs to only pest management professionals as well as continued public outreach through state wide Integrated Pest Management programs may be warranted. In addition, promotion of compounds that do not possess the propensity for secondary poisoning (i.e. zinc phosphide) should be considered in non-professional use settings. Furthermore, ARs in these habitats may pose equally grave risks to other rare and isolated California carnivores such as the Sierra Nevada red fox (*Vulpes vulpes necator*), American marten (*Martes americana*), wolverine (*Gulo gulo*), gray wolf (*Canis lupus*) or raptors such as northern spotted owls (*Strix occidentalis caurina*), California spotted owls (*S.o. occidentalis*) and great gray owls (*Strix nebulosa*). Future research should be directed to investigating potential risks to prey populations as well as other sympatric species that may allow a better understanding of the potential AR sources contributing to these exposure and mortality rates from anticoagulant rodenticides.

## Supporting Information

Table S1 **A two-way ANOVA analyzing the effects of California fisher (Martes pennanti) populations and sex on the number of anticoagulant rodenticides found per individual.**
(DOCX)

Table S2 **Results of spatial scan statistics to detect clusters of anticoagulant rodenticide (AR) exposed fishers within each California fisher project.** Number of individual fisher minimum convex polygon (MCP) centroids used for each temporal period, specific AR types, generation class of AR and distribution of numbers of ARs per fisher (number of AR positive fishers per test in parentheses) are shown.
(DOCX)
