# Supplementary material for: Anticoagulant Rodenticides on our Public and Community Lands: Spatial Distribution of Exposure and Poisoning of a Rare Forest Carnivore
Source: PLoS One. 2012 Jul 13;7(7):e40163. doi: 10.1371/journal.pone.0040163 (PMC3396649; doi:10.1371/journal.pone.0040163)
Supplement: Table S1 — A two-way ANOVA analyzing the effects of California fisher (Martes pennanti) populations and sex on the number of anticoagulant rodenticides found per individual. (DOCX) [file pone.0040163.s001.docx]

**Table S1: A two-way ANOVA analyzing the effects of California fisher (*Martes pennanti*) populations and sex on the number of anticoagulant rodenticides found per individual.**

| Variable | DF | Sum of Squares | Mean Squares | F ratio | Probability Level |
| --- | --- | --- | --- | --- | --- |
| Fisher Population (A) | 1 | 1.452 | 1.452 | 1.50 | 0.225 |
| Sex (B) | 1 | 1.335 | 1.335 | 1.38 | 0.245 |
| Fisher Population*Sex (AB) | 1 | 0.214 | 0.214 | 0.22 | 0.640 |
