# Supplementary material for: Anticoagulant Rodenticides on our Public and Community Lands: Spatial Distribution of Exposure and Poisoning of a Rare Forest Carnivore
Source: PLoS One. 2012 Jul 13;7(7):e40163. doi: 10.1371/journal.pone.0040163 (PMC3396649; doi:10.1371/journal.pone.0040163)
Supplement: Table S2 — Results of spatial scan statistics to detect clusters of anticoagulant rodenticide (AR) exposed fishers within each California fisher project. Number of individual fisher minimum convex polygon (MCP) centroids used for each temporal period, specific AR types, generation class of AR and distribution of numbers of ARs per fisher (number of AR positive fishers per test in parentheses) are shown. (DOCX) [file pone.0040163.s002.docx]

**Table S2: Results of spatial scan statistics to detect clusters of anticoagulant rodenticide (AR) exposed fishers within each California fisher project.** Number of individual fisher minimum convex polygon (MCP) centroids used for each temporal period, specific AR types , generation class of AR and distribution of numbers of ARs per fisher (number of AR positive fishers per test in parentheses) are shown.

| CA Fisher Project | Complete  MCP Centroids | Six-month  MCP Centroids | Three-month  MCP centroids | Individual AR | AR Generation | Multinomial | Monte-Carlo  Test statistic | Probability Level |
| --- | --- | --- | --- | --- | --- | --- | --- | --- |
| HVRFP | 12 (8) | 8 (6) | 11(8) | BRD: 12 (8) | 1^st^ generation: 12 (2)  2^nd^ generation: 12 (8) | Multi: 12 | Complete: 3.600  6-month: 1.726  3-month: 2.287  BRD: 2.100  1^st^: 1.633  2^nd^: 2.100  Multi: 6.279 | 0.620  0.765  0.600  0.198  0.860  0.198  0.102 |
| SNAMP | 19 (18) | 19 (18) | 19 (18) | BRD: 19 (17)  BRM: 19 (10)  DIP:19 (3)  CHL: 19 (2) | 1^st^ generation : 19 (4)  2^nd^ generation : 19 (17) | Multi: 19 | Complete: 0.667  6-month: 0.667  3-month: 0.667  BRD: 1.361  BRM: 2.22  DIP: 5.692  CHL: 2.257  1^st^: 3.991  2^nd^: 1.361  Multi: 5.839 | 1.000  1.000  1.000  0.810  0.940  0.108  0.680  0.368  0.810  0.790 |
| KRFP | 11 (9) | 4 (4) | 9(6) | BRD: 11 (9)  BRM: 11 (2)  DIP:11 (2) | 1^st^ generation : 11 (2)  2^nd^ generation : 11 (9) | Multi: 11 | Complete: 1.368  3-month: 2.316  BRD: 1.368  BRM: 3.239  DIP: 3.239  1^st^: 3.239  2^nd^: 1.368  Multi: 6.244 | 0.860  0.550  0.860  0.570  0.570  0.570  0.860  0.160 |
